# Comment on “Clinical management implications from 33,579 urinary stones: novel patterns in composition, comorbidities, seasonal variation, and machine learning-based urosepsis prediction”

**DOI:** 10.1097/JS9.0000000000004680

**Published:** 2026-01-20

**Authors:** Bangbei Wan, Weiying Lu

**Affiliations:** a Reproductive Medical Center, Hainan Women and Children’s Medical Center, Haikou, China; b Department of Urology, Haikou Affiliated Hospital of Central South University Xiangya School of Medicine, Haikou, China

*To the Editor,*

Chang *et al* report an impressive single-center series of 33 579 urinary stones collected over a decade in Southern China, providing a panoramic view of stone composition, comorbidity patterns, seasonal variation, and a machine learning (ML)-based model for urosepsis prediction^[1]^. In an era where urolithiasis is increasingly recognized as a systemic disease rather than an isolated urological problem, this work is timely and clinically relevant. In preparing this commentary, the authors adhered to the TITAN 2025 guidelines on transparency and integrity in the use of AI-assisted tools in surgical research reporting^[2]^.

Several elements of this study deserve emphasis. First, the authors confirm that calcium-containing stones remain predominant, but move beyond simple compositional reporting to link stone type with >30 comorbidities, including metabolic, cardiovascular, endocrine, and anatomical conditions^[1]^. They show that women have higher rates of infection stones, urinary tract infection, connective tissue disease, and anemia, whereas men more commonly harbor calcium stones, hyperuricaemia, gout, and cardiovascular disease^[1]^. These findings reinforce the European Association of Urology guidelines’ emphasis on metabolic and systemic evaluation, and suggest that sex-specific risk profiling should be more explicitly integrated into guideline-based pathways^[3]^.

Second, the authors elegantly characterize anatomical and seasonal heterogeneity. Calcium stones dominate in ureteral locations, uric acid stones are over-represented in bladder stones, and infection stones cluster in posterior urethral and renal calculi, with bladder stone patients showing a distinctly older, comorbidity-rich profile (high rates of benign prostatic hyperplasia, chronic obstructive pulmonary disease and cardiovascular disease)^[1]^. Seasonal analysis reveals summer peaks for infection stones and urinary tract infection, winter peaks for cardiovascular comorbidities, and autumn predominance for metabolic disorders such as hyperuricemia and hyperlipidemia^[1]^. These multidimensional patterns, rarely captured in prior epidemiological work, highlight that stone type, location, age, sex, and season jointly shape risk rather than acting in isolation.

Third, and most novel, is the development of an ML-based urosepsis prediction model. Using a 60:40 train–validation split and a consensus feature-selection framework (Lasso, XGBoost, logistic regression), the authors identify key predictors including sex, urinary tract infection, anemia, staghorn calculi, renal insufficiency, and urinary tract malformation, achieving an AUC of 0.802 in the validation set^[1]^. This aligns with a growing ML literature aiming to anticipate sepsis after endourological procedures and percutaneous nephrolithotomy, and supports the concept that data-driven models can complement clinical judgment for high-risk stone patients^[4–6]^.

However, several limitations should temper immediate clinical adoption. The retrospective, single-center design limits generalizability beyond a humid subtropical, East Asian context. Analysis is conducted at the stone level, with multiple stones per patient included; without accounting for within-patient clustering, effect estimates may be overly precise. Dietary intake, fluid consumption, body mass index, and medication use – strong determinants of stone risk – were unavailable. Urosepsis, though catastrophic, remained rare in this cohort, leading to substantial class imbalance and potential overfitting of the ML model despite internal validation. External validation in independent datasets, calibration assessment and decision-curve analysis will be essential before embedding this tool into routine practice.

From a management perspective, the sex-, age-, season-, and location-specific insights support more nuanced risk stratification. Men with metabolic syndrome and elderly males with uric acid bladder stones may benefit from intensified metabolic work-up and lifestyle interventions, while women with infection stones, recurrent urinary tract infections and systemic inflammatory or autoimmune disease may require closer infectious surveillance and earlier intervention. The strong association of staghorn and infection stones with urosepsis risk is consistent with previous reports and underscores the need for aggressive infection control and careful perioperative planning in these patients^[7]^.

In summary, Chang *et al* deliver the largest FTIR-based stone composition analysis reported from Asia, and uniquely integrate comorbidity profiling, environmental context and ML-based risk prediction^[1]^. Their work advances our understanding of how systemic health, season and stone characteristics intersect, and offers a promising – though still preliminary – framework for urosepsis risk stratification. Future studies should validate and refine these models across diverse populations and care settings, ideally within prospective, guideline-embedded pathways, to determine whether such tools can truly improve outcomes for patients with urinary stone disease.

## Data Availability

No data were used in this Letter to the Editor.
